# Evaluation of breastfeeding and infant feeding attitudes among syrian refugees in Turkey: observations of Syrian healthcare workers

**DOI:** 10.1186/s13006-023-00579-9

**Published:** 2023-08-09

**Authors:** Siddika Songül Yalçın, Meryem Erat Nergiz, Suzan Yalçın

**Affiliations:** 1 Departmant of Social Pediatrics, Faculty of Medicine, Institute of Child Health, Hacettepe University, Ankara, Turkey; 2 Department of Pediatrics, Yenimahalle Research Hospital, Yıldırım Beyazıt University, Ankara, Turkey; 3 Department of Food Hygiene and Technology, Faculty of Veterinary Medicine, Selçuk University, Konya, Turkey

**Keywords:** Syrian refugees, Breastfeeding, Social determinants of health, Breastfeeding-friendly, Turkey

## Abstract

**Background:**

The influx of Syrian refugees into Turkey has highlighted the importance of supporting breastfeeding practices among this vulnerable population. We aimed to evaluate the breastfeeding and infant feeding attitudes of Syrian mothers based on the observations of Syrian healthcare workers (HCWs).

**Methods:**

An online form including 31 questions was prepared in Turkish, Arabic, and English languages and distributed to HCWs, working in refugee health centers via e-mail, WhatsApp, or text message with the help of Ministry of Health in Turkey between January 2020 and March 2020. The questions were about HCWs’ characteristics (occupation, region of employment, duration of employment, participation in breastfeeding counseling course) and about HCWs’ observations of Syrian mothers’ breastfeeding and infant feeding practices.

**Results:**

A total of 876 HCWs were included in the study; about 37.3% were physicians. Only 40.0% of HCWs reported that babies were predominantly fed with breast milk in the first three days after birth, 45.2% of HCWs indicated that mothers typically used sugary water as a prelacteal food, and 30.5% believed that breastfeeding was discontinued before 12 months. The main barriers to breastfeeding identified by HCWs included the lack of education, mental and physical health issues in the mother, food insecurity, low income, inadequate housing, lack of family planning, sociocultural environment, and limited access to quality health services. For complementary feeding, 28.0% of HCWs stated early introduction and 7.4% remarked delayed. HCWs believed grains, fruits and vegetables, and dairy products as top three foods for starting complementary food (59.5%, 47.8%, and 30.3% respectively). Healthcare challenges of Syrian pregnant and lactating mothers were reported to be associated primarily with “food, finance, and housing difficulties”, low maternal education, and cultural and environmental issues. HCWs recommended various solutions, such as supporting breastfeeding, offering nutrition and health support, promoting family planning, improving healthcare systems through legislation, and addressing cultural barriers.

**Conclusions:**

To address breastfeeding issues among Syrian mothers, it is crucial to provide breastfeeding training to both HCWs and mothers. Expanding interventions that support breastfeeding-friendly practices, including community support and food aid for breastfeeding mothers, should also be considered to address the social determinants of breastfeeding.

## Background

Given the importance of breastfeeding for both mothers and infants, World Health Organization (WHO) recommends initiating breastfeeding in the first hour, exclusive breastfeeding for the first six months of life, followed by continued breastfeeding alongside complementary foods for up to two years and beyond [1, 2]. This recommendation is particularly crucial in high-risk situations as disasters or migration, where access to clean water is limited and the risk of infection is high, making artificial feeding more dangerous for child health and survival [3].

Since the onset of the Syrian war in 2011, Turkey has become the host country for a significant number of Syrian refugees due to its proximity to Syria. As of 2022, there are over 3.5 million Syrian refugees under temporary protection in Turkey [4]. Although breastfeeding is a fundamental right of the child, many factors determine the initiation and continuation of breastfeeding. Forced migration is known to have a negative impact on breastfeeding practices [5, 6]. Despite having a positive attitude towards breastfeeding, many Syrian refugee mothers have a shorter duration of breastfeeding, with most discontinuing before the child reaches 12 months [7, 8].

To address the healthcare needs of Syrian refugees, the SIHHAT project, funded by the European Union, has been implemented in Turkey. This project involves the establishment and expansion of refugee health centers (RHCs) that provide primary healthcare services to Syrian refugees under temporary protection. Syrian healthcare workers, including physicians and nurses, are employed in these centers [9].

This study aims to explore the observations and recommendations of Syrian healthcare workers (HCWs) working in RHCs regarding breastfeeding practices and feeding difficulties faced by Syrian refugee infants according to HCWs’ job and working region. By obtaining insights from these HCWs, it is possible to identify key areas for intervention and support to improve breastfeeding rates and address the challenges specific to this vulnerable population.

## Methods

### Study design

The study design of this research was a descriptive study conducted between January 2020 and March 2020 in collaboration with the Ministry of Health of Turkey and Hacettepe University. The study aimed to gather information about the observations of HCWs regarding breastfeeding and the nutritional status of Syrian refugee infants living in Turkey.

### Study population

The population of the study consisted of Syrian physicians and nurses (midwives were also considered in this category) working in RHCs in Turkey. HCWs of other nationalities and non-medical staff (translators, cleaning staff, etc.) were excluded from the study.

### Data collection

An online survey was created in Turkish, Arabic, and English languages and distributed to HCWs working at RHCs via email, WhatsApp, or short message service. HCWs were asked to choose and fill out the survey form in the language they were most comfortable with. The survey consisted of 31 questions, including the five questions about HCWs’ characteristics (occupation, region of employment, duration of employment, participation in breastfeeding counseling course) and 26 questions about HCWs’ observations of Syrian mothers’ breastfeeding and infant feeding practices. The survey included open-ended questions (*n* = 14). The Arabic survey forms were translated from Turkish by bilingual Syrian translators and back-translated into Turkish for accuracy. The same process was applied by the researchers for the English survey forms. At the beginning of the survey, 10 forms in each language were filled by the health personnel, the incomprehensible questions were reviewed, and the study form was rearranged.

Qualitative data obtained through Arabic forms were translated by bilingual translators. All qualitative data were grouped appropriately. Care was taken to create a large number of groups in order to avoid data loss and to fully understand the subject. For the same purpose, the proportions of the answers forming the groups were given as a separate table.

### Statistical analysis

The data were analyzed using the IBM-SPSS 23.0 program (SPSS Inc., Chicago, IL). The Kolmogorov-Smirnov test was used to check for normal distribution of data. Descriptive statistics were used to present continuous variables as mean ± standard deviation and categorical variables as frequency and percentage. Chi-square test or Fisher’s Exact test was used for comparisons between categorical variables. Subgrup analysis of proportions according to regions was performed using residual analysis with the Chi-square test. A significance level of *p* < 0.05 was used.

## Results

A total of 1161 survey forms were collected. Forms filled by HCWs of different nationalities or non-medical staff were excluded. In the end, 876 survey forms were suitable for analysis. However, 12 forms had been left blank in the question of the place of employment and the analyzes for working region were made in 864 survey forms.

The results of the study showed that out of the 876 Syrian HCWs included in the study, 37.3% were physicians and 62.7% were nurses or midwives. Most of the HCWs had been working for more than one year (45.4%). A small percentage (14.5%) preferred to fill out a Turkish survey form, and only 12% had attended a breastfeeding counseling course. The distribution of HCWs varied across different regions, with the highest proportion in the south (40.2%) and the lowest in the central region (9.4%) (Table 1).

**Table 1:** HCWs’ observations for breastfeeding characteristics of Syrian refugees

	Total	HCWs’ Job, %^a^		Working Region, %^a^			
	%^a^	Physician	Nurse	West	South	Central	East
Overall, n	876	327	549	199	352	82	231
(%)^b^		(37.3)	(62.7)	(22.7)	(40.2)	(9.4)	(26.4)
**Mothers, usually observed to give only breast milk to their babies in the first three days**	40.0	**34.6**	**43.9** ^*^	**51.8** ^**x**^	**35.5** ^**y**^	**52.4** ^**x**^	**32.5** ^y***^
**Prelacteal foods usually observed to be given**							
Sugary water	45.2	50.5	42.1	**32.7** ^**x**^	**49.7** ^**y,z**^	**39.0** ^**x,y**^	**51.9** ^z***^
Dairy products	8.7	10.1	7.8	9.0	8.2	9.8	9.1
Herbal tea	3.2	3.1	3.3	**2.5** ^**x,y**^	**2.3** ^**x**^	**1.2** ^**x,y**^	**6.1** ^y*^
Pure water	3.0	1.8	3.6	2.0	3.1	2.4	3.9
Sweet food	2.5	2.8	2.4	3.5	2.0	6.1	1.3
**Usually observed the initiation of breastfeeding within one hour**							
Frequently	91.6	**86.5**	**94.7** ^***^	**93.4** ^**x**^	**94.0** ^**x**^	**93.9** ^**x**^	**85.7** ^y**^
**Reasons for not achieving the initiation of breastfeeding within one hour**							
Not feeling well physically or mentally after delivery	8.6	**14.1**	**5.3** ^***^	5.5	8.8	11.0	10.0
Lack of experience or education	5.3	7.0	4.2	3.5	4.5	8.5	6.9
The belief of there is not enough breast milk	3.3	3.7	4.0	3.5	3.1	1.2	6.5
Illness of the mother or baby	3.0	4.0	2.4	1.5	3.1	6.1	2.6
**Usually observed exclusive breastfeeding duration**							
≥6 months	49.2	**35.9**	**57.1** ^***^	52.0	45.7	54.9	50.0
4–5 months	40.8	**52.8**	**33.7** ^***^	37.4	44.3	34.1	40.4
First 3 months	10.0	11.3	9.2	10.6	10.0	11.0	9.6
**Opinion for the reason why exclusive breastfeeding is shorter than six months**							
Breast milk insufficiency or breastfeeding problems	37.2	40.4	35.3	34.2	37.2	56.1	33.3
Factors related to maternal health	20.7	**26.0**	**17.5** ^**^	**22.1** ^**y**^	**22.4** ^**y**^	**32.9** ^**z**^	**12.1** ^x***^
Lack of experience or education	10.3	16.8	6.4	10.6	10.2	12.2	9.5
Frequent pregnancy plan	10.0	8.0	11.3	**4.0** ^**x**^	**16.8** ^**y**^	**8.5** ^**x,y**^	**5.6** ^x***^
Mother’s job-economic problems	4.7	**7.6**	**2.9** ^**^	**4.5** ^**x**^	**7.1** ^**x**^	**2.4** ^**x,y**^	**1.3** ^y**^
Culture-social environment	3.3	**93.9**	**98.4** ^***^	4.5	2.0	6.1	2.6
Illness of children	3.2	2.1	3.8	2.0	5.1	3.7	1.3
**Usually observed total breastfeeding duration**							
<6 months	10.5	8.6	11.7	**9.6** ^**x,y**^	**9.1** ^**x,y**^	**3.7** ^**x**^	**15.2** ^y*^
<12 months	30.5	28.1	31.9	29.1	30.4	23.2	34.2
**Usually observed infants receiving formula in the first six months**							
More than half	27.4	25.3	28.6	32.7	24.3	21.0	29.1
**Usually observed complementary feeding starting time**							
<6 months	28.0	**38.2**	**21.9** ^***^	30.7	30.7	26.8	22.1
≥9 months	7.4	**4.9**	**8.9** ^*^	8.5	7.4	7.3	6.9
**Foods that are usually observed to be started first in complementary feeding**							
Grains	59.5	61.5	58.3	**51.8** ^**x**^	**59.1** ^**x,y**^	**62.2** ^**x,y**^	**65.8** ^y*^
Fruits and vegetables	47.8	49.8	46.6	48.2	49.4	51.2	44.6
Dairy products	30.3	27.5	31.9	**23.6** ^**x**^	**33.0** ^**y**^	**40.2** ^**y**^	**29.0** ^x,y*^
Soups-snacks	9.0	7.0	10.2	6.5	7.4	12.2	12.1
Egg	4.6	3.4	5.3	5.0	4.8	4.9	3.9

^a^Column percentage; ^b^Row percentage; Comparison according to HCWs’ job and working region;  *p < 0.05, **p < 0.01, ***p < 0.001; The letters x,y,z indicate the statistically significant difference between the subgroups of the working region

HCWs’ observations for breastfeeding characteristics of Syrian refugees were given in Table 1 and breastfeeding problems in Syrian refugees in Table 2. Regarding breastfeeding practices, 40% of HCWs reported that babies were fed only with breast milk in the first three days after birth. HCWs stated the highest use of prelacteal foods such as sugary water in the East region and the lowest usage in the West (*p* < 0.001, Table 1). HCWs mentioned the highest frequency of herbal tea usage in the East (*p* < 0.05). Reported prelacteal foods by HCWs are seen in Table 3.

**Table 2:** HCWs’ observations for breastfeeding problems in Syrian refugees

	Total	HCWs’ Job, %^a^		Working Region, %^a^			
	%^a^	Physician	Nurse	West	South	Central	East
Overall, n	876	327	549	199	352	82	231
(%)^b^		(37.3)	(62.7)	(22.7)	(40.2)	(9.4)	(26.4)
**Are breastfeeding difficulties common according to HCWs observations?**							
Yes	26.5	**31.6**	**23.4** ^**^	24.4	24.1	28.0	29.0
**Rate of overcoming breastfeeding difficulties according to HCWs observations**							
More than half	70.7	70.6	70.7	**71.8** ^**x**^	**73.9** ^**x**^	**55.6** ^**y**^	**71.9** ^x*^
**Most common observed difficulties associated with breastfeeding according to HCWs observations**							
Lack of experience or education	10.7	12.2	9.8	9.0	9.4	13.4	13.0
Maternal-related factors	9.5	9.8	9.3	10.6	8.2	12.2	9.1
Lactation-related problems	5.8	7.6	4.7	6.0	4.3	6.1	7.8
Economic problems	5.4	7.6	4.0	5.5	6.8	3.7	3.0
Culture-related problems	3.2	4.3	2.6	3.0	2.8	6.1	2.6
Factors related to family planning	3.0	4.0	2.4	**1.5** ^**x**^	**3.7** ^**x,y**^	**7.3** ^**y**^	**1.7** ^x*^
**From whom do lactating mothers usually get support in baby feeding according to HCWs observations?**							
Mothers of her or her husband	69.1	69.9	68.9	68.3	71.3	74.4	64.9
Health care professionals	32.3	30.9	33.2	30.7	34.4	32.9	32.0
Other people	17.0	78.3	85.8	21.1	15.3	23.2	13.9
Husbands	9.7	89.0	91.1	13.1	8.0	13.4	8.7
**When getting pregnant again while breastfeeding**							
Usually breastfeeding is stopped	68.6	**60.0**	**73.8** ^***^	69.5	70.4	57.3	70.5
**How long does breastfeeding usually continue while pregnant according to HCWs observations?**							
Discontinued immediately or in the first trimester	71.9	69.1	73.6	69.8	74.4	64.6	73.2
Discontinued in the 2nd trimester	11.4	11.3	11.5	9.0	11.6	18.3	11.3
Discontinued in the 3rd trimester	2.3	2.4	2.2	2.0	2.6	1.2	2.2
Depending on the age of the breastfed child	6.7	8.3	5.8	7.5	5.7	8.5	6.1
Other	6.7	1.5	0.9	2.0	0.9	2.4	0.4
Missing	6.5	7.3	6.0	9.5	4.8	4.9	6.9
**Is tandem breastfeeding common according to HCWs observations?**							
Yes	29.6	32.0	28.2	29.7	28.7	24.7	33.0
**Healthcare challenges of Syrian pregnant and lactating mothers posed by HCWs**							
Food-finance-housing-related problems	34.9	36.4	34.1	**28.1** ^**x**^	**42.9** ^**y**^	**32.5** ^**x,y**^	**29.0** ^x**^
Low maternal education	27.3	29.7	25.9	**25.1** ^**x,y**^	**31.0** ^**y**^	**31.7** ^**y**^	**21.2** ^x*^
Cultural and environmental issues	19.6	19.6	19.7	**14.6** ^**x**^	**20.5** ^**x,y**^	**29.3** ^**y**^	**19.0** ^x,y*^
Maternal health-related problems	18.6	18.0	18.9	16.1	20.5	14.6	18.6
Problems with family planning	15.4	16.2	14.9	**10.1** ^**x**^	**15.1** ^**x**^	**30.5** ^**y**^	**15.6** ^x***^
Incorrect nutrition practices	8.9	9.5	8.5	8.0	9.1	8.9	9.6
Breastfeeding-related problems	5.5	5.5	5.5	5.0	6.0	1.2	6.9
Lack of health care	4.8	4.9	4.7	3.5	6.8	6.1	2.6
**Recommendations of HCWs to solve the difficulties experienced by Syrian mothers**							
Supporting breastfeeding	57.2	58.4	56.5	50.8	59.4	57.3	59.7
Nutrition, health support to the mother	35.7	67.6	62.3	28.1	39.5	34.1	36.8
Family planning	12.7	13.8	12.0	**11.1** ^**x,y**^	**14.8** ^**y,z**^	**23.2** ^**z**^	**7.8** ^x**^
Regulating the health system-legislation	8.6	**11.6**	**6.7** ^*^	8.0	6.5	11.0	10.8

^a^Column percentage; ^b^row percentage; Comparison according to HCWs’ job and working region: *p < 0.05, **p < 0.01, ***p < 0.001; The letters x,y,z indicate the statistically significant difference between the subgroups of the working region.

**Table 3:** **Prelacteal feeding and complementary feeding characteristics of Syrian refugees according to HCWs’ observation**

What foods are given to the baby in the first three days after birth, other than breast milk according to HCWs’ observation?	n (%)	With which foods do mothers start complementary feeding, according to HCWs’ observation?	n (%)
Sugary water	396 (45.2)	Grains	521 (59.5)
Dairy products	76 (8.7)	• Rice or rice flour	350 (40.0)
• Milk	57 (6.5)	• Wheat flour or wheat starch	116 (13.2)
• Formula	16 (1.8)	• Baby food with grain	89 (10.2)
• Marjujah (goat milk with tea)	2 (0.2)	• Biscuit	12 (1.4)
• Yogurt	1 (0.1)	• Bread	4 (0.5)
Herbal tea	28 (3.2)	• Oat	2 (0.2)
• Anise tea	24 (2.7)	Fruits and vegetables	419 (47.8)
• Cumin tea	4 (0.5)	• Fruit puree or boiled fruit	280 (32.0)
• Mint tea	2 (0.2)	• Boiled vegetables	177 (20.2)
• Herbal tea	2 (0.2)	• Banana	51 (5.8)
Plain water	26 (3.0)	• Fruit juice	50 (5.7)
• Plain water	25 (2.9)	• Tomato juice	6 (0.7)
• Zamzam (sacred water)	1 (0.1)	• Avocado	1 (0.1)
Sweet food	22 (2.5)	Dairy products	265 (30.3)
• Date	9 (1.0)	• Milk	172 (19.6)
• Fruit	6 (0.7)	• Yoghurt	105 (12.0)
• Honey	3 (0.3)	Soups-snacks	79 (9.0)
• Turkish delight	2 (0.2)	• Soups	72 (8.2)
• Grape molasses	1 (0.1)	• Snacs	7 (0.8)
		Egg	40 (4.6)
		Flesh foods	5 (0.5)
		• Red meat	3 (0.3)
		• White meat	2 (0.2)
		Unsuitable foods	8 (0.9)
		• Tea bread mix	3 (0.3)
		• Coffee	3 (0.3)
		• Honey	2 (0.2)

HCWs stated “the initiation of breastfeeding within one hour” for most Syrian mothers (Table 1). They cited “Not feeling well physically or mentally after delivery” as the most common reason (8.6%) for not achieving the initiation of breastfeeding within one hour. Almost half of those was “maternal mental illness” (3.8%) (Table 4). The initiation of breastfeeding within one hour was reported less frequently in the East region and among physicians (Table 1). More than one-third HCWs remarked “Breast milk insufficiency or breastfeeding problems” as the first common reason for shorter breastfeeding duration (Table 1). One-fifth stated “Factors related to maternal health” as the second reason in which the rates of statement were changed according the working region and profession of HCWs; highest in Central Region and Physicians questionaries. The answer “Factors related to maternal health” included not only physical but also mental health (Table 4). “Lack of experience or education” and “Frequent pregnancy plan” were the other frequent reasons stated by HCWs (Table 4).

**Table 4:** Breastfeeding barriers according to HCWs’ observations

What is the reason why babies cannot be breastfed within the first hour after birth?	N (%)	The reason why exclusive breastfeeding is shorter than six months	N (%)
**Not feeling well physically or mentally after delivery**	75 (8.6)	**Breast milk insufficiency or breastfeeding problems**	326 (37.2)
• Mother’s postpartum fatigue and pain	42 (4.8)	• Insufficient breast milk production	244 (27.9)
• Mother’s mental illness	33 (3.8)	• Insufficient weight gain of the child	34 (3.9)
• Nipple pain	2 (0.2)	• Frequent crying / not getting enough of the baby	33 (3.8)
**Lack of experience or education**	46 (5.3)	• Believing that breast milk is not enough	32 (3.7)
• Mothers do not know how to breastfeed and are inexperienced	41 (4.7)	• Breast problems	16 (1.8)
• Unable to attach baby to breast	6 (0.7)	• Breast rejection	7 (0.8)
**The belief of there is not enough breast milk**	29 (3.3)	• Artificial feeding	5 (0.6)
• Failure of milk to reach the breast	32 (3.7)	• Twin birth	4 (0.5)
• Baby’s crying or not getting enough	1 (0.1)	**Factors related to maternal health**	181(20.7)
**Illness of the mother or baby**	26 (3.0)	• Maternal poor health/drug use	107 (12.2)
**Delivery type**	19 (2.2)	• Maternal malnutrition	79 (9.0)
• Cesarean delivery	16 (1.8)	• Mother’s mental illness	15 (1.7)
• General anesthesia given during delivery*	6 (0.7)	• Aesthetic concerns	5 (0.6)
**Cultural misconception**	13 (1.5)	• Desire to gain weight	3 (0.3)
**Deficiencies in Baby-Friendly Hospital Initiative implementation**	3 (0.3)	• Absence of mother	2 (0.2)
• Uneducated health professionals	1 (0.1)	**Lack of experience or education**	90 (10.3)
• The impact of formula companies in the hospital	1 (0.1)	• Not knowing the importance of breast milk, no education	77 (8.8)
• Doctors recommend formula	1 (0.1)	• Mother’s inexperience	13 (1.5)
		• Lack of health care	6 (0.7)
		**Frequent pregnancy plan**	88 (10)
		**Mother’s working conditions-economic problems**	41 (4.7)
		• Mother’s work	27 (3.1)
		• Economic problems	15 (1.7)
		**Culture-social environment**	29 (3.3)
		**Illness of children**	28 (3.2)

*The belief that breastfeeding should not be done immediately after cesarean section because the mother has taken medication

HCWs reported that exclusive breastfeeding duration was less than six months in more than half of Syian infants and total breastfeeding duration was less than six months in one-tenth (Table 1). The total breastfeeding duration less than six months was stated highest in the east region and the lowest in the central region (15.2%, 3.7%, respectively).

Overall, 28.0% of HCWs stated early introduction of complementary feeding and 7.4% remarked delayed starting for complementary feeding. Early introduction of complementary feeding was reported more by physicians, whereas delayed introduction for complementary feeding by nurses (*p* < 0.001, *p* < 0.05; respectively). About 27.4% of the HCWs said that they usually observed that more than half of the mothers gave formula in the first six months (Table 1).

HCWs believed grains, fruits and vegetables, and dairy products as top three foods for starting complementary food (59.5%, 47.8%, and 30.3% respectively). The belief of HCWs was changed with working region for grains and dairy products (Table 1). A small number of HCWs (0.9%) said that mothers usually gave unsuitable foods (tea bread mix, coffee and honey) for complementary feeding (Table 3).

A quarter of HCWs stated breastfeeding difficulties as a common problem (Table 2). More physicians than nurses stated breastfeeding difficulties as a common problem (31.6%, 23.4%, *p* < 0.01). Overcoming more than half problems were reported in 70.7% of HCWs and this was similar in physicians and nurses. HCWs in the Central Region reported the lowest success rate for the management of the half of breastfeeding problems (55.6%).

“Lack of experience or education”, “ Maternal-related factors”, “Lactation-related problems”, and “Economic problems” were the top four difficulties (10.7%, 9.5%, 5.8%, and 5.4%, respectively) associated with breastfeeding according to HCWs utterance (Table 2). According to 69.1% of HCWs, grandmothers were identified as the primary source of support for lactating mothers. The majority of HCWs (68.6%) reported that breastfeeding was immediately stopped when the mother became pregnant, and only 29.6% of HCWs reported that tandem feeding was practiced (Table 2).

Healthcare challenges of Syrian pregnant and lactating mothers posed by HCWs were given in detail in Table 5. “Food-finance-housing-related problems” (34.9%), “low maternal education” (27.3%) and “cultural and environmental issues” (19.6%), maternal health-related problems (18.6%), and challenges for family planning (15.4%) were frequently observed problems related to healthcare and nutritional characteristics of pregnant women and mothers (Tables 2 and 5). Some observations were changed according to region; utterence for finance problems mostly reported in South region, low maternal education in South and Central region. In addition, Central region reported more cultural and environmental problems and problems with family planning (Table 2).

**Table 5:** Healthcare challenges of Syrian pregnant and lactating mothers posed by HCWs according to HCWs’ observations

Challenges	N(%)
**Food-finance-housing-related problems**	**306 (34.9)**
• Malnutrition of the mother or child	200 (22.8)
• Financial deficiencies/poor living conditions	115 (13.1)
• Mother’s work	34 (3.4)
• Expensive, paid/not-free formulas	4 (0.5)
**Low maternal education**	**239 (27.3)**
**Cultural and environmental issues**	**172 (19.6)**
• Ignoring recommendations/not attending following up	113 (12.9)
• Cultural barriers	73 (8.3)
• Adolescent marriage/adolescent pregnancy	61 (7.9)
• Tetanus vaccine refuse (in pregnancy)	6 (0.7)
• Divorces/marriage problems	5 (0.6)
• Unsafe abortions	1 (0.1)
**Maternal health-related problems**	**163 (18.6)**
• Maternal poor health/drug use	56 (6.4)
• Psychological problems	50 (5.7)
• Maternal anemia	40 (4.6)
• Poor hygiene of mother and baby	25 (2.9)
• Not wanting to breastfeed	8 (0.9)
• Aesthetic concerns	2 (0.2)
• Not drinking a lot of water	1 (0.1)
**Challenges for family planning**	**135 (15.4)**
• Short pregnancy interval/getting pregnant while breastfeeding	124 (14.2)
• Absence or refusal of family planning	11 (1.3)
• Leaving breastfeeding to get pregnant again	2 (0.2)
**Incorrect mothers’ nutrition practices**	**78 (8.9)**
• Starting complementary feeding early	19 (2.2)
• Unnecessary/inappropriate formula feeding	18 (2.1)
• Negligence of baby	12 (1.4)
• Concerns about breast milk’s sufficient and baby growth	11 (1.3)
• Breastfeeding while pregnant	8 (0.9)
• Early weaning	8 (0.9)
• Not breastfeeding immediately after birth	4 (0.4)
• Feeding baby with cow milk	3 (0.3)
• Pacifier use	3 (0.3)
• Starting complementary feeding late	2 (0.2)
• Trying to lose weight while breastfeeding	2 (0.2)
• Not breastfeeding while pregnant	1 (0.1)
• Late weaning	1 (0.1)
• Cessation breastfeeding due to jaundice	1 (0.1)
**Breastfeeding-related difficulties**	**48 (5.5)**
• Breast problems	37 (4.2)
• Insufficient breast milk	14 (1.6)
**Lack of health care**	**42 (4.8)**
• Insufficient pregnant-baby follow-up/difficulty of reaching health centers	39 (4.5)
• Mothers who do not have IDs do not apply to health centers.	3 (0.3)

Details of recommendations of HCWs to solve the difficulties experienced by Syrian mothers were seen in Table 6. More than half of HCWs recommended “supporting breastfeeding” to solve the difficulties experienced by Syrian mothers (Tables 2 and 6). Nearly one-third recommended “nutrition and health support to the mother”. More than one-tenth of HCWs recommended “family planning” to solve the difficulties. This recommendation was most expressed in the Central region (23.2%) and less stated in the East region (7.8%) (*p* < 0.01; Table 2). Compared to nurses, more physicians recommended “Regulating the health system legislation” (*p* < 0.05).

**Table 6:** Recommendations of HCWs to solve the difficulties experienced by Syrian mothers according to HCWs’ observations

Recommendations	N (%)
**Supporting breastfeeding**	**501 (57.2)**
• Educating breastfeeding mothers	457 (52.2)
• Solving breast problems	34 (3.9)
• Initiating breastfeeding immediately after birth	21 (2.4)
• Frequent breastfeeding	20 (2.3)
• Cleaning or wiping the breast	20 (2.3)
• Providing formula to mothers who cannot breastfeed	1 (0.1)
**Nutrition and health support to the mother**	**313 (35.7)**
• Feeding mothers well, ensuring adequate nutrition of the mother	241 (27.5)
• Psychological support for mothers	54 (6.2)
• Vitamin supplement for mother	53 (6.1)
• Increasing family income / providing food aid	40 (4.6)
• Encouraging mothers to breastfeed	25 (2.9)
• Mothers should pay attention to cleanliness	20 (2.3)
• Arrangement of working hours of mothers	7 (0.8)
• Reducing odors and deodorants	1 (0.1)
**Family planning**	**111 (12.7)**
**Regulating the health system-legislation**	**75 (8.6)**
• Periodic follow-up of mothers and babies	49 (5.6)
• Educating healthcare personnel	12 (1.4)
• Formula sales must be by prescription or warnings should be written on the boxes	8 (0.9)
• To open comprehensive and widespread health centers	3 (0.3)
• Information through the media	2 (0.2)
• Researching on problems	2 (0.2)
• Providing convenience to nurses	1 (0.1)
• Providing breastfeeding rooms	1 (0.1)
**Overcoming cultural barriers**	**15 (1.7)**
• Prevention of adolescent marriages	10 (1.1)
• Overcoming language and culture barriers	5 (0.6)

HCWs identified various healthcare challenges faced by Syrian pregnant and lactating mothers, including food, finance, and housing-related problems, low maternal education, cultural and environmental issues, and challenges related to family planning (Table 5). Recommendations to address these difficulties included supporting breastfeeding, providing nutrition and health support to mothers, and promoting family planning (Table 6). Trained HCWs who had attended a breastfeeding counseling course had different perspectives compared to their counterparts in some areas, such as the role of culture and social environment, maternal-related factors, and paternal support.

## Discussion

According to the observations of HCWs, Syrian mothers usually start breastfeeding within the first hour, breastfeed their babies exclusively for the first six months, and stop breastfeeding after 12 months in our study. However, more than half of HCWs said mothers usually give prelacteal foods and nearly one-third said mothers start complementary foods before six months. According to the TDHS 2018 Syrian sample, 73% of children started breastfeeding within the first hour, 24% received prelacteal foods, and the frequency of exclusive breastfeeding was 6% for infants aged 6–8 months [10]. Previously, a study conducted in Turkey revealed that the rate of exclusive breastfeeding ≥ 6 months in Syrian babies was 28.1%, the rate of the initiation of breastfeeding within one hour was 61.4%, and all the breastfeeding indicators in Syrian refugees were lower than that for local women in Turkey [5]. In a study conducted in Lebanon, the percentages of prelacteal feeding, the initiation of breastfeeding within one hour, and the exclusive breastfeeding were 62.5%, 31%, and 24.6% respectively, in Syrian refugees [11]. It was seen that HCWs in this study perceived breastfeeding rates higher than they were in previous surveys. As shown, the breastfeeding attitudes of Syrian refugees vary according to the country and region where the study is conducted. There are also regional differences in this study.

Most of the HCWs said prelacteal feeding was common among Syrian refugees. As a prelacteal food, the HCWs observed sugary water usage more common in the south and east regions closer to the Syrian border than in the western region far from the border. In addition, milk, formula, and herbal teas were stated as prelacteal food. In a study conducted in Jordan, it was found that 64.3% of Syrian refugee mothers gave prelacteal foods to their babies; of them, water (99.7%), sugary water (64.5%), milk-formula (52.7%), and herbal tea (29.3%) were most frequently given [12]. In a qualitative study conducted in Turkey, most Syrian mothers said they gave prelacteal foods such as sugary water, packaged fruit juice, infant formula, anise, dates, honey, cumin, and Zamzam (religiously blessed plain water) [6].

HCWs identified maternal health conditions, including malnutrition, as the primary barriers to initiating breastfeeding within one hour and exclusive breastfeeding. Half of the HCWs believed that supporting mothers, and one out of every three said that providing them with nutritional and health support would help address breastfeeding problems. The study also highlighted the impact of social determinants of health, such as food insecurity, maternal physical and mental health, family income, housing, education, working conditions, and the sociocultural environment, on breastfeeding attitudes and practices. According to a qualitative study conducted in Turkey, Syrian refugees think that breastfeeding negatively affects maternal health and that lactating mothers should be well-fed [6]. In a mixed-method study conducted with Syrian refugees in Lebanon, maternal health was defined as one of the barriers to breastfeeding [11]. In a qualitative study conducted in eastern Uganda, women highlighted hunger as a cause of insufficient milk production [13]. A study conducted in South Africa found that mothers living in low socioeconomic conditions and experiencing hunger breastfeed less frequently [14]. According to a study conducted in Kenya, it was predicted that women living in houses with no food security would not be able to breastfeed their babies exclusively in the first six months, women who gave only breast milk for six months would experience health or social problems, and women would need sufficient food to support breastfeeding [15]. The maternal experience of hunger can contribute to perceived milk insufficiency, anxiety about infant hunger, and a perception that access to adequate food is necessary for successful breastfeeding [13–15]. Therefore, breastfeeding support should include nutritional and economic support for the mother. In addition, household food insecurity was among the situations that limited breastfeeding in many studies [16–20].

Maternal mental health is reported as a barrier to breastfeeding in our study. Some studies reported that postpartum depression and anxiety were associated with the discontinuation of breastfeeding, especially in refugees [6, 21, 22]. In a recent systemic review, 12 of 33 studies reported significant positive effects of behavioral interventions on maternal mental health and breastfeeding success [23]. On the other hand, a previous study showed a significant relationship between maternal symptoms of mental health problems and breastfeeding self-efficacy [24], showing the importance of multi-dimensional interaction [6, 25].

Overall, HCWs have associated the mother’s breastfeeding status with factors such as the mother’s physical and mental health, family income, housing, education, working conditions, inability to access quality health care, and socio-cultural environment. All these factors are also social determinants of health [26]. As a result of studies conducted in the United States, education, employment, food, neighborhood, housing, family income, and discrimination have been defined as social determinants of breastfeeding, and interventions targeting these determinants have been proposed to improve breastfeeding rates [27].

The Baby Friendly Hospital Initiative is the principal program to support breastfeeding and is based on the Ten Steps [28]. Nine of these steps involve hospital practices, while step 10 (Community Support) is about maintaining breastfeeding support after discharge from the maternity hospital. Step 10 appears to be key for the long-term sustainability of the short-term breastfeeding gains obtained due to the Baby Friendly Hospital Initiative efforts focusing solely on maternity facilities. Facilities providing maternity and newborn services need to identify appropriate community resources for continued and consistent breastfeeding support that is culturally and socially sensitive to their needs. These include guidance in primary healthcare centers, mother-to-mother support, family support, and advertisement of breastfeeding [6, 29–32]. Therefore, extending step 10 to target the social determinants of breastfeeding should be considered. In Turkey, primary health care is provided to refugees in RHCs. Only 12% of the HCWs in these centers received breastfeeding counseling training during the study period. With the results of this study, breastfeeding counseling training for HCWs was initiated in RHCs.

The study acknowledged some limitations. The study was conducted in RHCs in Turkey, and the selection of Syrian HCWs was not randomized, and may not be generalizable to other settings or populations. The study relied on personal observations of HCWs and provided only second-hand information. Also, most of the HCWs did not have breastfeeding counseling courses, and the adequacy of trained HCWs was controversial. Some HCWs may be woefully ignorant about infant feeding, subject to recall or social desirability bias. As a limitation of the self-administered survey, since these statements were not followed up and controlled in the study design, it is unknown whether the health professionals solve these problems, which problems, and to what extent they solve them. This study was not conducted directly with mothers and focused on the perspectives of HCWs, limiting a comprehensive understanding of mothers’ experiences and attitudes toward breastfeeding. But the fact that the included HCWs were from the same community. The study was conducted within a specific timeframe and may not capture the long-term trends or changes in breastfeeding practices among Syrian refugee mothers. Despite these limitations, the study emphasized the need for supporting breastfeeding among vulnerable populations like refugees. This study can be both a stepping stone to future qualitative studies and a guide to interventions that target the social determinants of breastfeeding in refugees.

## Conclusions

According to HCWs observations, prelacteal feeding and giving sugary water within 2–3 days after birth are quite common among Syrian refugees, and the most important barriers to breastfeeding are thought to be the lack of education, poor mental and physical health of the mother, food insecurity, low income, housing, lack of family planning, sociocultural environment and the inability to access quality health services. In order to increase breastfeeding rates in refugees, intervention programs including the social determinants of breastfeeding should be developed and integrated into Step 10 of the Baby Friendly Hospital Initiative, especially food aid to breastfeeding mothers, training of primary healthcare workers, and increasing the quality of care.

## Data Availability

The data of this study are available from the corresponding author upon reasonable request.
